# Effects of High-Temperature Annealing Atmosphere on the Secondary Recrystallization Behavior and Magnetic Properties of Fe-3.2%Si-0.055%Nb Grain-Oriented Silicon Steel

**DOI:** 10.3390/ma15238388

**Published:** 2022-11-25

**Authors:** Liguang Wang, Shuhuan Wang, Jie Li, Minghe Zhang, Yunli Feng

**Affiliations:** College of Metallurgy and Energy, North China University of Science and Technology, Tangshan 063009, China

**Keywords:** 0.055% Nb grain-oriented silicon steel, annealing atmosphere, secondary recrystallization, texture, magnetic property

## Abstract

High-temperature annealing is a key step for the secondary recrystallization of grain-oriented silicon steel, which has an important effect on the final sharp Goss texture. In this work, the effects of three different annealing atmospheres during high-temperature annealing (100%H_2_, 25%N_2_ + 75%H_2_ and 50%N_2_ + 50%H_2_) on the secondary recrystallization microstructure and texture of Fe-3.2%Si-0.055%Nb low temperature grain-oriented silicon steel were analyzed by optical microscopy (OM) and electron back-scattered diffraction (EBSD) techniques, and the magnetic properties of the samples were compared. The results show that when the high-temperature annealing atmosphere is 100%H_2_, the texture of the grains with secondary recrystallization is mainly <110>//ND orientation, but the grains without secondary recrystallization have a disordered orientation. When the high-temperature annealing atmosphere is 50%H_2_ + 50%N_2_, the secondary recrystallization grains present a Goss texture and brass texture. After high-temperature annealing in the 25%N_2_ + 75%H_2_ atmosphere, the sample can be fully recrystallized to obtain secondary recrystallization grains; the grain size is relatively uniform and the texture is mainly a Goss texture with a sharp edge. The content of this reaches 93.1%, the magnetic induction B_800_ is the highest, reaching 1.89 T, and the iron loss P_1.7/50_ is the lowest, reaching 1.33 W/kg.

## 1. Introduction

Grain-oriented silicon steel is an indispensable soft magnetic material in electronics, electrical and military fields. It is widely used in various transformers and large generator cores because of its excellent magnetic properties [1,2,3]. The key technology of grain-oriented silicon steel production is to use fine and dispersed inhibitors to inhibit the normal growth of primary recrystallization grains, and to develop secondary recrystallization to obtain a sharp Goss texture by using the surface energy difference in the grains with different orientations [4,5,6]. In traditional grain-oriented silicon steel production, MnS and AlN + MnS are mainly used as inhibitors, which have a high solution temperature. Its slab heating temperature is as high as 1673 K, which could cause problems such as a high consumption, a large surface burning loss and an increased production cost [7,8]. In terms of the problems mentioned above, developing new inhibitors or adjusting the composition of the existing inhibitors to reduce the reheating temperature of the slab and meet the high-quality requirements of steel has become a research hotspot in the grain-oriented silicon steel production industry. Previous studies have shown that [9,10,11,12] Nb, as a strong carbonitride forming element, is easy to form carbides, nitrides and carbonitrides in grain-oriented silicon steel, which has a low solid solution temperature; moreover, the precipitated particles are uniform and fine, the coarsening rate is low and it has a strong inhibitory effect. It has the general characteristics of being an inhibitor of grain-oriented silicon steel and can achieve the purpose of reducing the reheating temperature of a slab. The results show that the solution temperature of NbC is 1250 °C and that of NbN is 1350 °C. Using their lower solution temperature can effectively reduce the slab heating temperature. Nb can not only refine the primary recrystallized grains during the intermediate annealing process, making them finer and more uniform, but also effectively improve the grain distribution of the intermediate annealing plate. In addition, Nb also plays a very significant role in increasing the strength of the {111}<112>texture. In the process of high-temperature annealing, various inhibitors of Nb formation can not only effectively inhibit the growth of normal grains, but also make Gaussian grains lose their pinning to grow abnormally and increase the sharpness of the Gaussian texture. High-temperature annealing is a key step for the secondary recrystallization of grain-oriented silicon steel, which has an important effect on the final sharp Goss texture [13,14,15,16,17]. During high-temperature annealing, the ripening behavior of the inhibitor in the secondary recrystallization stage is adjusted by changing the ratio of N_2_ and H_2_ in the protective atmosphere, so as to promote the full growth of Goss-oriented grains and inhibit the growth of other oriented grains [18,19]. The results show that [20] the pinning ability of the inhibitor decreases faster in a high H_2_ atmosphere, resulting in larger primary recrystallization grains, a faster growth rate and a shorter duration of secondary recrystallization. The deflection Goss-oriented grains grow, and the secondary recrystallization is incomplete. In contrast, in a high N_2_ atmosphere, due to the slow decline of the pinning ability of the inhibitor, the primary recrystallization grain is small, the growth rate is slow, the duration of secondary recrystallization is prolonged, the abnormal growth of deflection Goss-oriented grains is restrained, the Goss-oriented grain grows abnormally and the secondary recrystallization is complete. Therefore, researching the composition of the atmosphere during high-temperature annealing is of great significance for improving the magnetic properties of low-temperature grain-oriented silicon steel and for revealing the formation mechanism of Goss grains during the secondary recrystallization.

The change in the atmosphere ratio during the high-temperature annealing will affect the inhibitor distribution and transformation in the grain-oriented silicon steel, which will further affect the abnormal growth of the Goss grains. It is generally considered that the appropriate high-temperature annealing atmosphere is related to the chemical composition of its main inhibitors [16,21]. At present, there are many studies on the effect of a high-temperature annealing atmosphere on the Hi-B grain-oriented silicon steel with AlN and MnS as inhibitors. By contrast, there are few reports on the evolution law of the microstructure and texture of the low-temperature grain-oriented silicon steel with Nb(C,N) + AlN as the main inhibitor during the secondary recrystallization process. In this work, by adjusting the ratio of N_2_ and H_2_ in the protective atmosphere during the high-temperature annealing of Nb-containing low-temperature grain-oriented silicon steel, the effects of high-temperature annealing in different atmospheres on the secondary recrystallization structure, texture and magnetic properties of such steel were investigated. The research results provide a theoretical basis for obtaining better magnetic properties in practical applications.

## 2. Materials and Methods

**Figure 1:**
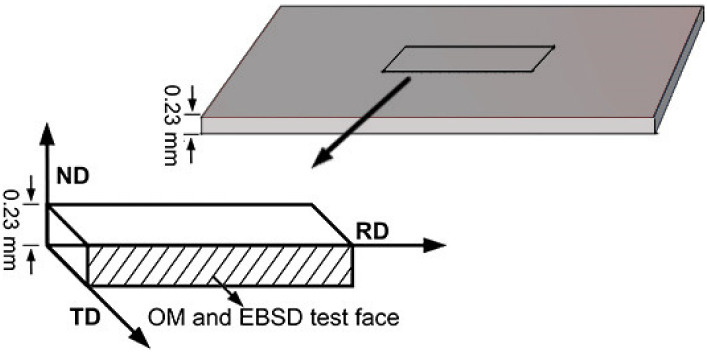
Schematic diagram of sampling position and the observation plane NDxRD.

The experimental material was smelted in a 50 kg vacuum induction furnace and then cast into a rectangular slab with a size of 240 mm × 90 mm × 30 mm. The chemical composition is shown in Table 1. The as-cast billets were, respectively, heated to 1220 °C, held for 30 min and then hot rolled to a thickness of 2.3 mm through a mill with 350 mm diameter rolls. The secondary cold rolling process is adopted for cold rolling; the total reduction was 88%. The reduction of the first cold rolling was 70%. After 4 passes of rolling, a 0.69 mm thick cold rolled sheet is obtained. After intermediate annealing, the second cold rolling was carried out. The reduction in the second cold rolling was 60%, and a 0.23 mm thick cold rolled sheet was obtained through three passes of rolling in a 4 h rolling mill of Φ170 mm × 250 mm. After hot rolling, normalizing, cold rolling and decarburization annealing, the slab was annealed at a high temperature in a vacuum/atmosphere tubular electric furnace (RTP-G06123K-H, Tianjin Zhonghuan Electric Furnace, Tianjin, China). In the high-temperature annealing process, the pre-treated samples were put into the furnace, heated to 1200 °C at a heating rate of 50 °C/h and three different atmospheres (100%H_2_, 25%N_2_ + 75%H_2_ and 50%N_2_ + 50%H_2_) were introduced during the annealing process. We then switched to a 100%H_2_ atmosphere, and the temperature was kept for 15 h, then cooled to 600 °C at a cooling rate of 50 °C/h. Finally, we switched to 100%N_2_ and they were cooled down to room temperature with the furnace. After being annealed at a high temperature, the samples were cut into 10 mm × 5 mm rectangular samples with a wire cutting machine. The sampling position and the observation plane NDxRD is shown in Figure 1. After grinding and polishing, the samples were corroded with 4% nitric acid alcohol solution for 13 s, and then a Zeiss microscope (Axio observer, Zeiss, Dresden, Germany) was used to observe the microstructure. After the samples were electropolished, a thermal field emission scanning electron microscope (Quanta-650FEG, FEI, Hillsboro, OR, USA) and EBSD system were used to analyze the texture type, texture content, orientation difference and grain size of the samples. The accelerating voltage was 20 kV, the working distance was 14~18 mm and the scanning step size was 1.5 µm. A soft magnetic AC measuring instrument (MATS-2010SA, Hunan Linkjoin Technology, Loudi, China) was used to test the magnetic properties of the final grain-oriented silicon steel product.

## 3. Results and Discussion

### 3.1. Effect of High-Temperature Annealing Atmosphere on the Secondary Recrystallization Microstructure

**Figure 2:**
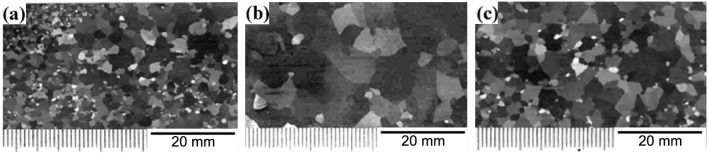
Macrostructure of finally annealed samples in different atmospheres: (**a**) 100%H_2_; (**b**) 25%H_2_ + 75%N_2_; (**c**) 50%N_2_ + 50%H_2_.

**Figure 3:**
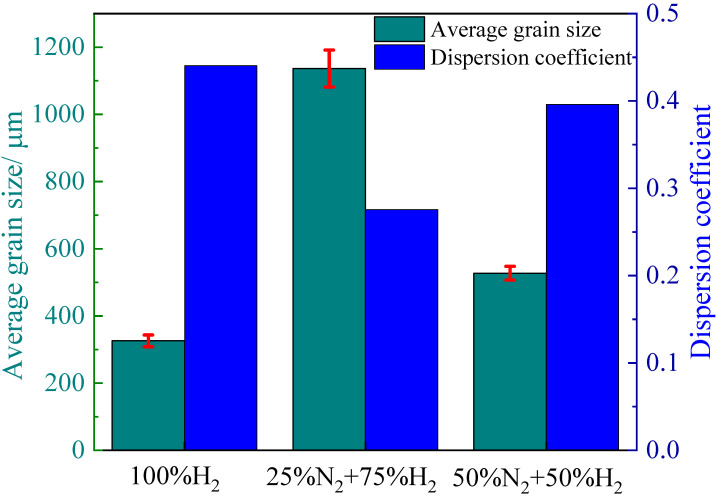
Diagram of average grain size and dispersion coefficient.

Figure 2 shows the macrostructure of the samples after the high-temperature annealing. Figure 3 shows the average grain size and dispersion coefficient. It can be seen from Figure 2 that the samples undergo different degrees of secondary recrystallization under different annealing atmospheres, the grain boundaries of the secondary recrystallization grains are bent and folded and there are island grains less than 3 mm in diameter distributed in the large grain boundaries or in the crystals. From Figure 2a, when the high-temperature annealing atmosphere is 100% H_2_, the microstructure of the finished grain-oriented silicon steel does not undergo a complete secondary recrystallization; there are a large number of relatively small primary recrystallization grains among the abnormally grown secondary recrystallization grains, and a certain degree of normal grain growth occurs, with the average grain size being 325 μm. This is because the partial pressure of N_2_ was too low during the high-temperature annealing process, and the free nitrogen in the silicon steel was precipitated, which is not conducive to the formation of inhibitors such as AlN, Nb(C,N), etc. This will weaken the pinning effect of the inhibitors on the primary recrystallization grains so that the primary recrystallization grains are easily separated from the inhibitor pinning and grow up. The larger the size of the primarily recrystallization grains, the smaller the driving force of the secondary recrystallisation, which makes it difficult for the abnormally grown Goss grains to swallow the primarily recrystallization grains, resulting in an incomplete secondary recrystallisation. In addition, the higher the amount of H_2_ in the high-temperature annealing atmosphere, the lower the secondary recrystallization temperature and the lower the growth rate of the secondary recrystallization grains [16]. The secondary recrystallization grains have grown up before they can swallow the primary recrystallization grains, thus the secondary recrystallization is difficult to continue. Therefore, an incomplete secondary recrystallization occurred. From Figure 2c, when the high-temperature annealing atmosphere was 50%N_2_ + 50%H_2_, the finished silicon steel basically underwent a secondary recrystallization, but the grain size was very uneven. This is mainly due to the high nitrogen content in the annealing atmosphere, which forms a large number of inhibitors with a strong inhibition ability, which inhibits the abnormal growth of the Goss grains to a certain extent. With the increase in the secondary recrystallization temperature, the inhibitors coarsen rapidly at a high temperature, which results in the abnormal growth of the Goss texture, deflection Goss texture, brass texture and other near Goss-oriented grains, affecting the magnetic properties of the final product. According to Figure 2b, when the high-temperature annealing atmosphere is 25%N_2_ + 75%H_2_, the finished silicon steel undergoes a complete secondary recrystallization; the secondary recrystallization grains have a uniform size distribution, the grain size difference is small and the average grain size is 1 cm. The appropriate ratio of nitrogen and hydrogen will make N_2_ and H_2_ react with Fe as the medium in a high-temperature environment: 3H_2_ + N_2_ = 2NH_3_. The generated NH_3_ decomposition will increase the N content in the silicon steel, forming a certain amount of small second-phase particles like AlN and Nb(C,N). These inhibitors can inhibit the growth of the primary recrystallization grains and make the secondary recrystallization grains easier to swallow these relatively small primary recrystallization grains, and thus abnormal growth occurs. Finally, the secondary recrystallization grains are well developed. The uniformity of the grains can be expressed by the dispersion coefficient. From Figure 3, when the high-temperature annealing atmosphere is 100% H_2_ and 50% N_2_ + 50% H_2_, the grain dispersion coefficient values are large, 0.44 and 0.39, respectively, which means that the difference between most grain sizes of the sample and its average grain size is large, indicating that the microstructure’s uniformity is poor at this time. When the high-temperature annealing atmosphere is 25% N_2_ + 75% H_2_, the value of the grain dispersion coefficient is the smallest, only 0.27, which shows that the sample has the best microstructure uniformity, and its average grain size is also the largest.

### 3.2. Effect of High-Temperature Annealing Atmosphere on the Secondary Recrystallization Texture

**Figure 4:**
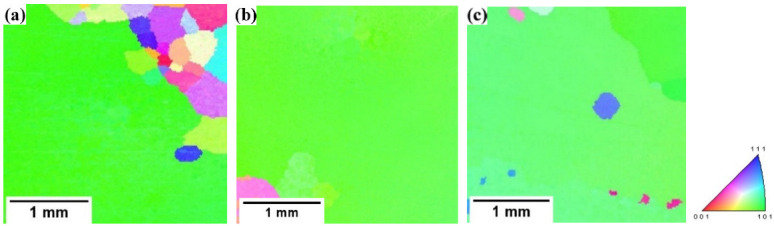
IPF of finally annealed samples in different atmospheres: (**a**) 100%H_2_; (**b**) 25%H_2_ + 75%N_2_; (**c**) 50%N_2_ + 50%H_2_.

Figure 4 shows the IPF images of the samples annealed at a high temperature in different atmospheres. In the figure, the red represents the texture of <001>//ND (normal direction), the blue represents the texture of <111>//ND and the green represents the texture of <110>//ND. It can be seen from Figure 4 that there are abnormally grown grains in the three samples, but the distribution of the microstructure and texture of the three samples is different. It can be seen from Figure 4a that when the high-temperature annealing atmosphere is 100% H_2_, the secondary recrystallization texture is mainly green <110>//ND orientation, but there are also many relatively small primary recrystallization grains that have not undergone a secondary recrystallization; their textures are of various orientations and these primary recrystallization grains have grown up and are difficult to be swallowed by the green secondary recrystallization grains. From Figure 4b, when the high-temperature annealing atmosphere is 25%H_2_ + 75%N_2_, the texture of the sample after high-temperature annealing is basically green <110>//ND texture. It can be concluded that the appropriate atmosphere composition plays an important role in the sharpness of the high-temperature annealing texture. It can be seen from Figure 4c that when the high-temperature annealing atmosphere is 50%H_2_ + 50%N_2_, the samples basically undergo a secondary recrystallization after the high-temperature annealing, but some near <111>//ND-oriented island grains still exist inside the secondary recrystallization grains. The secondary recrystallization texture is not a single green <110>//ND orientation, but there are also some near green secondary recrystallization textures. It can be seen that when the N_2_ content in the atmosphere increases, the sharpness of the secondary recrystallization texture decreases. This is mainly because with the increase in the N_2_ content, the inhibitor increases too much, the secondary recrystallization temperature increases and the inhibitor particles coarsen too fast at a high temperature, so that other oriented grains with a high grain boundary mobility, except a Goss texture, also grow abnormally, but these textures have an adverse impact on the magnetic properties of the final product.

**Figure 5:**
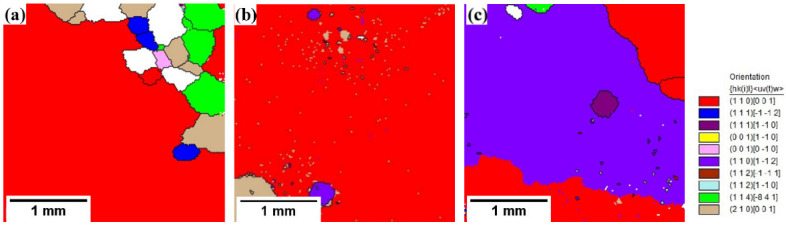
EBSD orientation maps of finally annealed samples in different atmospheres: (**a**) 100%H_2_; (**b**) 25%H_2_ + 75%N_2_; (**c**) 50%N_2_ + 50%H_2_.

Figure 5 shows the EBSD orientation images of the samples annealed at a high temperature in different atmospheres. It can be seen from Figure 5a,b that the grains which underwent a secondary recrystallization are all sharp Goss orientations, while Figure 5c is different. The secondary recrystallized grains not only contain Goss orientation, but also contain {110}<112> brass texture. According to Figure 5a, the orientations of some grains without a secondary recrystallization mainly included {210}<001>, {111}<112>, {411}<148> and {001}<010> textures. These grains are not swallowed by the huge Goss grains but coexist with the Goss grains. This is mainly due to the fact that the nitrogen potential of the sample surface is higher than that of the atmosphere during the high-temperature annealing process, the nitrogen atoms diffuse outward, the content of the inhibitors such as AlN and Nb(C,N) decreases, and the inhibitory force is insufficient. As a result, after the decarburization annealing process, many favorable textures such as {111}<112> and {411}<148> fail to be swallowed by Goss grains and instead they grow up in advance, and finally survive. The research has shown [6] that although there is a special orientation relationship between the {111}<112> texture and Goss texture, the {111}<112> primarily recrystallization grain size is too large to be swallowed by the Goss grain in a very fast manner. The {210}<001> oriented grains have a relatively low misorientation angle (about 20°) with the Goss orientation, which is difficult to be absorbed by the Goss grains. Meanwhile, the {110} <112> brass texture has a {110} low-energy crystal plane as the Goss texture, which is also prone to a secondary recrystallization during high-temperature annealing, and the grains have size advantages, so that they cannot be swallowed by Goss grains [22]. Grains with a smaller deviation angle from the ideal Goss direction have advantages in overcoming inhibitor pinning compared with grains with larger deviation angles [23]. From Figure 5b, almost all of the secondary recrystallization grains are Goss grains, and there are only a small amount of {110}<112> and {210}<001> oriented island grains. From Figure 5c, the {110}<112> brass texture also undergoes a secondary recrystallization: it competes with the Goss texture to grow up and is difficult to be swallowed, which has a negative effect on the magnetic properties of the final product. It is generally believed that the growth driving force of Goss grain is greater than that of brass grains. With the prolongation of the annealing time, the inhibitor gradually coarsens and the inhibitory force gradually decreases; when the inhibitory force decreases to the value of the driving force of the Goss grain growth, Goss grains preferentially swallow the surrounding small grains and grow up. When the inhibitory force decreases to the driving force of the brass grain growth, the grains also begin to grow up. In the 50%N_2_ + 50%H_2_ atmosphere, the inhibitor rapidly coarsens at a high temperature and the growth interval Δt between the brass grains and Goss grains decreases, which inhibits the growth of Goss grains and leads to the competitive growth between the brass grains and Goss grains.

Table 2 shows the content ratios of the several main textures of the samples annealed at a high temperature in different atmospheres. It can be seen from Table 2 that the final Goss texture content in the 25%N_2_ + 75%H_2_ atmosphere is the highest, reaching 93.1%; that of the samples annealed in the 100% H_2_ atmosphere is 78.1% and that of the samples annealed in the 50% N_2_ + 50% H_2_ atmosphere is the lowest at 27.9%. For the {110}<112> brass texture, its content obtained in the 50%N_2_ + 50%H_2_ atmosphere reached 70%; that obtained in the 25%N_2_ + 75%H_2_ atmosphere is 1.4% and that obtained in the 100%H_2_ atmosphere is only 0.1%. For the {210}<001> texture, its content obtained in the 100%H_2_ atmosphere is the highest, being 6.1%, and that obtained in the 25%N_2_ + 75%H_2_ atmosphere is 5.5%, with that obtained in the 50%N_2_ + 50%H_2_ atmosphere being only 0.1%. The {111}<112> and {411}<148> textures are mainly in the sample with the 100%H_2_ atmosphere, whose content is 1.9% and 6.7%, respectively, while the other two samples have very little content of these two textures. This is mainly because in the 100% H_2_ atmosphere, the inhibitory force is not enough, which makes the primary recrystallized grains easy to grow away from the inhibitor pinning. The larger the grain size of the primary recrystallization is, the smaller the driving force of the secondary recrystallization is, so that the abnormally grown Goss grains have no time to swallow the primary recrystallization grains. The primary recrystallization grains have grown, and the secondary recrystallization is difficult to continue. Therefore, the secondary recrystallization is not perfect. As a result, some {111}<112>and {411}<148> textures remain.

**Figure 6:**
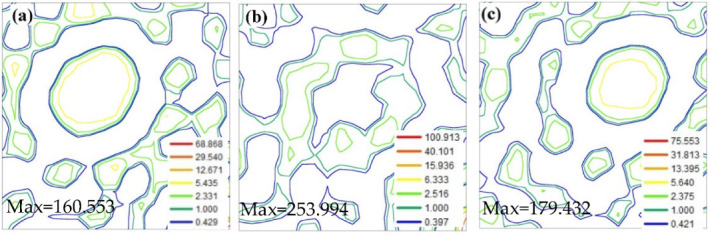
ODF sections (φ_2_ = 45°) of finally annealed samples in different atmospheres: (**a**) 100%H_2_; (**b**) 25%H_2_ + 75%N_2_; (**c**) 50%N_2_ + 50%H_2_.

Figure 6 is the cross section of the texture orientation distribution function φ_2_ = 45° of the samples annealed at a high temperature in different atmospheres. It can be seen from Figure 4 that only one of the textures is very sharp, while the other textures are relatively loose. From Figure 6a,b, the sharpest textures of the samples annealed in the 100%H_2_ atmosphere and 25%H_2_ + 75%N_2_ atmosphere are both Goss textures, and the maximum orientation density is 160.533 and 253.944, respectively. Obviously, the sharpness of the Goss texture of the sample annealed at a high temperature in the 25%H_2_ + 75%N_2_ atmosphere is higher. The difference between the two samples is that the sample obtained in the 100%H_2_ atmosphere has a sharper {411}<148> texture, while the sample obtained in the 25%N_2_ + 75%H_2_ atmosphere has more diffuse textures. This statistical result is consistent with the results in Table 2. It can be seen that the {411}<148> texture will break away from the inhibitor pinning and rapidly grow up when the inhibitor content is small and the inhibitory force is weak; finally, it is difficult to be swallowed by Goss grains due to the excessive grain size. This results in the appearance of a large number of relatively small primarily recrystallization grains after high-temperature annealing. The result of Figure 6c is obviously different from the former two. The sharpest texture is the {110}<112> brass texture, and the maximum orientation density is 179.432. In addition, the Goss texture is also relatively sharp. This indicates that the inhibitor rapidly coarsened during the high-temperature annealing process because the secondary recrystallization temperature is too high. The brass texture competed with the Goss texture to grow up, and the brass texture has an advantage in the abnormal growth process, but the brass texture’s orientation is not <110> in the easy-magnetization direction, which has a negative effect on the magnetic properties of the final product.

**Figure 7:**
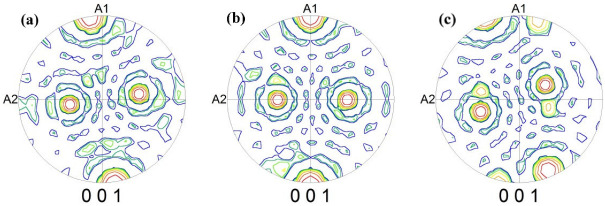
{001} pole figures of finally annealed samples in different atmospheres: (**a**) 100%H_2_; (**b**) 25%H_2_ + 75%N_2_; (**c**) 50%N_2_ + 50%H_2_.

Figure 7 shows the {001} pole figure of the samples annealed at a high temperature in different atmospheres. It can be seen from Figure 7 that the {001} pole figure of the samples annealed at a high temperature in different atmospheres have different degrees of sparseness and the orientation is also different. From Figure 7b, the Goss texture of the sample annealed at a high temperature in the 25%N_2_ + 75%H_2_ atmosphere is very sharp and the orientation is accurate. From Figure 7a, it can be seen that the texture orientation of the Goss texture in the sample obtained in the 100%H_2_ atmosphere is deviated and the deviation angle is large. Figure 7c is different from the former two, where the Goss texture has a low sharpness, the orientation deviation is large and a sharper brass texture appeared. This is very disadvantageous to the final product of the oriented silicon steel with a high magnetic induction and a low iron loss.

**Figure 8:**
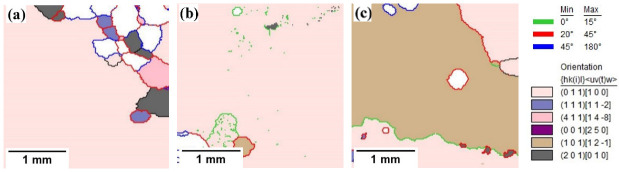
Grain boundaries of finally annealed samples in different atmospheres: (**a**) 100%H_2_; (**b**) 25%H_2_ + 75%N_2_; (**c**) 50%N_2_ + 50%H_2_.

Figure 8 presents the grain boundary distribution diagram of the samples annealed at a high temperature in different atmospheres. The figure shows the distribution of the grain boundaries of the samples annealed at a high temperature in different atmospheres, and the relationship between the grain boundaries and some main orientation grains. It can be seen from Figure 8 that the grain boundary compositions of the samples annealed at a high temperature in three different atmospheres included a high-energy grain boundary, a high-angle grain boundary and a small-angle grain boundary, but the proportions of the compositions are different. From Figure 8a, a large number of primary recrystallization grains that have not been swallowed penetrate deep inside the abnormally grown Goss grains. These grains mainly include {411}<148>, {111}<112> and {210 }<001> orientations, with a mainly high-energy grain boundary between these grains and Goss grains, and a small number of high-angle grain boundaries are also included. These grain boundaries have a higher energy and a higher migration speed. However, as the grain size has reached the limit where the secondary recrystallization grains can rapidly swallow them up, a large number of small grains with different orientations remained after the high-temperature annealing. By contrast, the phenomenon in Figure 8b is different from that in Figure 8a. Because the sample undergoes a complete secondary recrystallization after high-temperature annealing in the 25%N_2_ + 75%H_2_ atmosphere, the textures are composed of large Goss grains. However, there are some island-like grains inside the Goss grains and the grain boundaries between most of these island grains and Goss grains are a small angle grain boundary or a high-energy grain boundary. This is mainly because some grains have a small misorientation angle with the Goss grains, forming a small-angle grain boundary and making them difficult to be swallowed. Some grains are surrounded by a high-energy grain boundary, which also has the ability to grow preferentially. Finally, these grains cannot be swallowed by Goss grains and thus remain. It can be seen from Figure 8c that there is a low-angle grain boundary or a high-energy grain boundary between the competing growing grains of brass and Goss, and both of them have a low-energy {110} crystal planes. They grow up and contact with each other, and finally presented this result. Additionally, both kind of the grains are too large to swallow each other, so they finally exist in the matrix of the oriented silicon steel.

**Figure 9:**
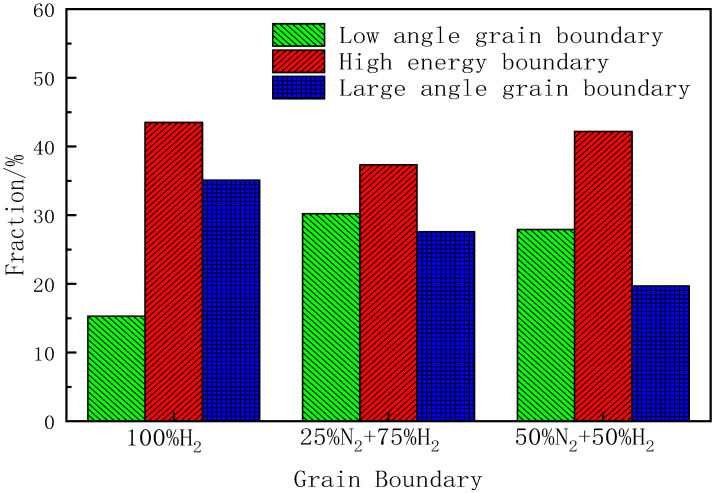
Grain boundaries content of finally annealed samples in different annealing atmospheres.

Table 3 shows the main grain boundary content of the samples annealed at a high temperature in different atmospheres. Figure 9 shows the content distribution of the main grain boundary of the samples annealed at a high temperature in different atmospheres. Combining Table 3 and Figure 9, it can be seen that when the annealing atmosphere is 100%H_2_, the content of the low-angle grain boundary is very small; the content of the high-energy grain boundary is much higher, with the content of 43.5%. This is mainly due to the lack of inhibition of the inhibitor during high-temperature annealing, which results in the growth of a large number of primary recrystallization grains with the textures such as {111}<112>, {411}<148>. These textures mainly form high-energy grain boundary with Goss textures. However, due to the reduction in the dislocation and sub-structure during high-temperature annealing, the content of the low-angle grain boundary is relatively low. When the annealing atmosphere is 25%H_2_ + 75%N_2_, the content of the low-angle grain boundary reached 30.2%; that of the high-energy grain boundary is 37.3% and that of the large-angle grain boundary is only 27.6%. This is mainly due to the complete secondary recrystallization of the samples in this atmosphere and the low grain boundary’s density. As a result, the grain boundaries between the island grains and the secondary recrystallization grains are mainly the low-angle and high-energy grain boundaries. When the annealing atmosphere is 50%N_2_ + 50%H_2_, the relative content trend of each type of grain boundary in this atmosphere is similar to that in the 25%N_2_ + 75%H_2_ atmosphere, and the formation reasons are also similar.

### 3.3. Effect of High-Temperature Annealing Atmosphere on Magnetic Properties of Finished Product

A soft magnetic AC measuring instrument (MATS-2010SA, Hunan Linkjoin Technology, China) was used to measure the magnetic induction intensity B_800_ and iron loss P_1.7/50_ of the finished oriented silicon steel sheet after high-temperature annealing in different atmospheres. The measurement results are shown in Table 4. It can be seen from Table 4 that the B_800_ value of the 25%N_2_ + 75%H_2_ atmosphere is the highest, reaching 1.89 T; that of the 100%H_2_ atmosphere is 1.76 T and that of the 50%N_2_ + 50%H_2_ atmosphere is the lowest, being 1.56 T. For the iron loss P_1.7/50_ value, it is the lowest in the 25%N_2_ + 75%H_2_ atmosphere, being 1.33 W/kg, followed by 1.41 W/kg in the 50%N_2_ + 50%H_2_ atmosphere, and it is the highest in the 100%H_2_ atmosphere, being 1.45 W/kg. The reason for this result is that the sample annealed at a high temperature in the 25%N_2_ + 75%H_2_ atmosphere undergoes a complete secondary recrystallization, and the abnormally grown grains are all sharp Goss textures. Due to the magnetocrystalline anisotropy in the crystalline magnetic material, the <100> crystal axis is the easy-magnetization direction, while the <111> crystal axis is the difficult-magnetization direction, and the other crystal axis directions are in between. As a result, the sharp Goss texture makes the final product have a high magnetic induction intensity, and the complete secondary recrystallization structure effectively reduced the hysteresis loss and the final iron loss. Due to the high sharpness of the Goss texture, the sample obtained in the 100%H_2_ atmosphere also has a high magnetic induction intensity, reaching more than 1.70 T. However, due to the mixed crystal phenomenon of the sample, the movement of the domain wall is hindered and the change in the magnetic flux is hindered, which leads to the increase in the hysteresis loss and the final iron loss [24]. For the sample obtained in the 50%N_2_ + 50%H_2_ atmosphere, the {110}<112> brass texture grows abnormally, the <112> crystal axis is not the easy-magnetizable direction and the sharpness of the Goss texture is low, which lowers the magnetic induction intensity of the final product. However, due to the small effect on the hysteresis loss, the iron loss is slightly lower than the sample obtained in the 100%H_2_ atmosphere.

## 4. Conclusions

(1) After high-temperature annealing in the 100%H_2_ atmosphere, the finished product of grain-oriented silicon steel do not undergo a complete secondary recrystallization, and there are many relatively small primarily recrystallization grains among the abnormally grown secondary recrystallization grains. After high-temperature annealing in the 50%N_2_ + 50%H_2_ atmosphere, the finished silicon steel basically undergoes a complete secondary recrystallization, but the grain size is very uneven. When annealed at a high temperature in the 25%N_2_ + 75%H_2_ atmosphere, the finished silicon steel undergoes a complete secondary recrystallization, the size of secondary recrystallization grains is evenly distributed and the average grain size reaches the centimeter level.
(2) When the high-temperature annealing atmosphere is 100%H_2_, the secondary recrystallization texture is mainly <110>//ND orientation, but the orientation of the grains without a secondary recrystallization is disordered. When the high-temperature annealing atmosphere is 25%H_2_ + 75%N_2_, the texture of the sample after the high-temperature annealing is basically all <110>//ND texture. When the high-temperature annealing atmosphere is 50%H_2_ + 50%N_2_, the secondary recrystallization texture is not a single <110>//ND orientation.
(3) When the high-temperature annealing atmosphere is 100%H_2_ and 25%H_2_ + 75%N_2_, the grains that underwent a secondary recrystallization are all sharp Goss orientations, with a content of 78.1% and 93.1%, respectively. When the high-temperature annealing atmosphere is 50%H_2_ + 50%N_2_, the secondary recrystallization grains have not only a Goss texture but also a brass texture; the content of the brass texture reaches 70%.
(4) The sample annealed at a high temperature in the 25%H_2_ + 75%N_2_ atmosphere has the best magnetic properties, with the highest magnetic induction intensity B_800_ of 1.89 T and the lowest iron loss P_1.7/50_ of 1.33 W/kg.

## Figures and Tables

**Table 1:** Chemical compositions of grain-oriented silicon steel (wt.%).

C	Mn	Si	S	Cu	Al	Nb	N	P
0.041	0.04	3.2	0.0075	0.085	0.023	0.055	0.0085	<0.008

**Table 2:** Content of main texture (/%).

Texture Content	{110}<001>	{110}<112>	{210}<001>	{111}<112>	{411}<148>
100%H_2_	78.1	0.1	6.1	1.9	6.7
25%H_2_ + 75%N_2_	93.1	1.4	5.5	0.00	0.00
50%N_2_ + 50%H_2_	27.9	70.0	0.1	0.05	0.4

**Table 3:** Content of main grain boundary (/ %).

Atmosphere	Grain Boundary Type		
	Low Angle Grain Boundary	High Energy Boundary	Large Angle Grain Boundary
100%H_2_	15.3	43.5	35.1
25%N_2_ + 75%H_2_	30.2	37.3	27.6
50%N_2_ + 50%H_2_	27.9	42.2	19.7

**Table 4:** Magnetic properties of grain-oriented silicon steel.

Magnetic Properties	100%H_2_	25%N_2_ + 75%H_2_	50%N_2_ + 50%H_2_
B_800_/T	1.76	1.89	1.56
P_1.7/50_/W/kg	1.45	1.33	1.41

## Data Availability

Data are available from the corresponding authors upon reasonable request.
